# Quackery Unmasked

**Published:** 1859-03

**Authors:** 


### QUACKERY UNMASKED.

Is the title of a dollar book by Dr. D. King, of Boston, which is alike suggestive to medical students, practising physicians, and all who think for themselves, which by the way is a very small army, but to be of that army an indispensible pre-requisite is, that a man must be the "bravest of the brave."

The Doctor wields a facile pen, and no reflecting mind will fail to be amused and instructed by the perusal of the work. And when we consider how many young physicians, on the advent of "medical inhalation," wrote patronising letters to its propagators, thereby showing their ignorance of medical history, and a consciousness of their incompetency to the scientific application of medical science—when this is brought to mind, we doubt not that if our medical schools would make Dr. King's book one of the standard works, a very salutary influence would be the result.

Dr. King wields a trenchant blade, and his cleaver of historical facts falls mercilessly on some of the isms of the day.—The closing paragraph in reference to women practising medicine, is characteristic of the whole book—"But when she enters the fetid laboratory of the anatomist, and plunges her hands into the gore of dead men, she loses all her feminine loveliness, and appears like a fallen angel, an object of universal horror and disgust."

More than one-half the book is devoted to the annihilation of Homeopathy. Its founder, a Dutchman, was born over a hundred years ago, and died at a good age. Dr. King's illustrations run about as follows: Hahneman's system was founded on two theories. The first was that "like cured like," that what causes a disease will cure it. If a man is sick at the stomach, give him an emetic. If another is going down hill give him a kick, and it will bring him back.

The other foundation stone of the system is, that if any medicine is valuable as a remedy, it becomes more powerful by division. That is, if a drop of cologne be put into a hundred drops of water, and be shaken a hundred times, it will have a hundred times more powerful effect, (will smell stronger we presume,) than it did at first; and that it may be gone on in this way until a drop will finally impregnate lake Superior, and that if one drop of this be taken, it will produce the most tremendous effects on the whole human frame, which will last a month. Hahneman carried this process through two thousand vials, and on giving a patient six or eight drops of it, he came very near killing him.

According to this, a grain of pulverized charcoal, divided into a million parts, will produce over two hundred symptoms of disease from the crown of the heel to the toe of the head; and that these symptoms will last thirty-six days. See "Jahr's new Homeopathic Manual," page 565.

The one decillionth part of a grain of common chalk gives a hundred and twenty-five diseased conditions of the body.

Professor Wharton, of London, says,—that one grain of medicine dissolved in a hundred drops of water, and a drop of that into a hundred other drops of water, until one drop of water has in it a decillionth part of the original grain, it would take a million of people a million of years, swallowing one drop a second, to take that grain of medicine; and that the vessel which should contain it all, would be a million miles long, a million miles broad and a million miles deep. We have not given the words of Dr. King exactly, but have given the ideas. If our infinitessimal friends think that we have not given a fair statement of the case, they must quarrel with their leaders, Hahneman, Jahr and others. If our readers do not desire to be bothered in the fog of the multitudinous pathies and isms of the times, we advise them to the use of natural inexpensive agencies, at least in the treatment of ordinary ailments, such as colds, neuralgias, dyspepsia, constipation, sick head ache, and the like; these agencies being a wise adaptation of food, rest, air, warmth, cleanliness and exercise, as uniformly and consistently set forth in our practice! as well as in our writings.

